# Beyond Hemostasis: Platelet Biomarkers and Vascular‐Immune Crosstalk in Dengue

**DOI:** 10.1096/fj.202600884RR

**Published:** 2026-06-22

**Authors:** Amanda Beatriz Adriano da Silva‐Alencar, Amanda de Oliveira Matos, Daniel Francisco de Sousa, Jefferson do Carmo Dietz, Eugenio Damaceno Hottz, Marcelle Figueira Marques da Silva‐Sales, Helioswilton Sales‐Campos

**Affiliations:** ^1^ Institute of Tropical Pathology and Public Health Federal University of Goiás Goiânia Goiás Brazil; ^2^ Federal University of Juiz de Fora Juiz de Fora Minas Gerais Brazil

**Keywords:** biomarkers, dengue, endothelial‐inflammatory axis, platelet immunity, thrombocytopenia

## Abstract

Dengue is an arboviral disease with major global public health impact. Its pathophysiology involves complex interactions between the virus and the host, including the immune response and the vascular endothelium. In this context, platelets play roles beyond hemostasis, acting as key immunomodulatory cells involved in inflammation and the regulation of vascular permeability. Therefore, this review summarizes and discusses the so called classic and non‐classic platelet‐related and endothelial biomarkers described in dengue virus infection. Classical biomarkers include P‐selectin (CD62P), PF4/CXCL4, RANTES/CCL5, CD40L, IL‐1β, VEGF, integrin αIIbβ3, CD63, and platelet extracellular vesicles, all supported by broad experimental and clinical evidence. Non‐classical or emerging biomarkers include TLT‐1, TREM‐1/sTREM‐1, angiopoietins (Ang‐1/Ang‐2), serotonin, ferritin, dengue virus envelope domain III (EIII), and soluble endothelial molecules such as IL‐1RA, sCD163, SDC‐1, sVCAM‐1, IL‐8, and IP‐10. Alone or in combination, these markers can be used/explored as prognostic tools and/or as therapeutic targets. Finally, integrating multimarker panels encompassing hemostatic, inflammatory, and endothelial pathways may improve prognostic stratification. This approach could support targeted interventions and help reduce complications such as thrombocytopenia, plasma leakage, and dengue shock.

## Graphical abstract

Several biomarkers are associated with platelet activation during dengue virus (DENV) infection. These include serum markers (AST, ALT, ferritin), platelet surface or secreted molecules (P‐selectin, PF4, RANTES, VEGF‐A, CD40L, TREM‐1, TLT‐1), immune and endothelial markers (TREM‐1, Ang‐1, Ang‐2), and viral components such as DENV EIII. While some are well‐established classical markers of platelet activation, others have been more recently proposed and still require further investigation, being considered non‐classical or potential markers.

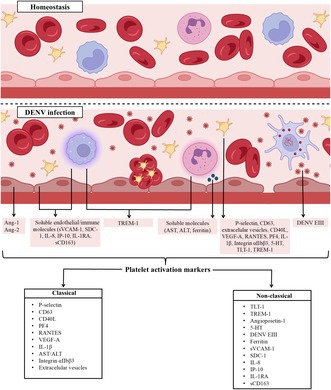

## 1 Introduction

Dengue represents one of the greatest challenges in global public health, especially in tropical and subtropical areas, and is often categorized as a neglected tropical disease [1, 2, 3, 4]. Its wide geographic distribution—encompassing Southeast Asia, the Western Pacific islands, Latin America, and Africa—makes it endemic in several regions, with estimates pointing to approximately 105 million infections per year with an annual global cost of around US$ 8.9 billion [5, 6, 7, 8]. The disease is primarily transmitted by the mosquito *Aedes aegypti* [9], and its spread is strongly linked to environmental and socioeconomic factors such as climate change, unplanned urbanization, and inadequate sanitation [10, 11].

The dengue pathophysiology involves complex interactions between the virus, the host (especially with the immune system), and the vascular microenvironment. Dengue virus (DENV) is a positive‐sense single‐stranded RNA virus belonging to the Flaviviridae family. Its genome encodes three structural proteins—envelope (E), premembrane/membrane (prM/M), and capsid (C)—and seven nonstructural proteins (NS1, NS2A, NS2B, NS3, NS4A, NS4B, and NS5). In addition, DENV comprises four antigenically distinct serotypes (DENV1–4). The viral structure consists of capsid, membrane, envelope, and viral genome [12, 13, 14, 15, 16]. Infection with one serotype generally confers long‐lasting immunity against that same serotype but not very effective against others. Reinfection with a heterologous serotype can trigger an exacerbated immune response, a phenomenon known as antibody‐dependent enhancement (ADE), which increases viral load and contributes to severe forms of the disease, such as dengue hemorrhagic fever (DHF) and dengue shock syndrome (DSS) [17, 18, 19]. These severe forms are characterized by plasma leakage, increased vascular permeability, and, in extreme cases, multiorgan failure [2, 20, 21].

In this context, platelets have emerged as key elements in the immune response and vascular pathophysiology during DENV infection. In addition to their classical role in hemostasis and thrombus formation [22, 23], platelets act as immune cells, releasing a variety of cytokines and growth factors, such as platelet factor 4 (PF4/CXCL4), regulated on activation normal T cell expressed and secreted (RANTES/CCL5), transforming growth factor‐beta (TGF‐β), and vascular endothelial growth factor (VEGF‐A), stored in their α‐granules. In addition, platelets contain dense granules enriched in small bioactive molecules, including adenosine diphosphate (ADP), adenosine triphosphate (ATP), serotonin, and calcium, which play key roles in platelet activation and inflammatory responses [24, 25, 26]. During dengue infection, excessive platelet activation may contribute to increased vascular permeability and plasma leakage, thereby negatively impacting clinical outcome [27]. Moreover, thrombocytopenia is a common clinical finding and is associated with disease severity, being used as a prognostic parameter [28, 29, 30]. So far, different mechanisms have been proposed to explain thrombocytopenia in DENV infection: platelet opsonization followed by macrophage‐mediated phagocytosis; DENV infection of megakaryocytes leading to their death and impaired platelet production; direct platelet infection, and platelet activation resulting in adhesion to the vascular endothelium and thrombus formation [31, 32, 33, 34].

Despite some existing discrepancies, the most consistent model of platelet infection suggests that platelets in contact with DENV exhibit an abortive infection phenotype, characterized by viral entry, limited RNA replication, and translation of viral proteins, but without efficient release of virions. In this regard, clinical and ex vivo evidence suggest that, although viral RNA synthesis and the production of proteins such as NS1 can be observed, DENV does not progress to a complete replicative cycle; therefore, suggesting that intrinsic limitations of the platelet machinery may prevent virion release [35, 36]. On the other hand, earlier studies have suggested that platelets may produce virions, particularly under in vitro conditions with purified and high DENV loads [37, 38, 39]. Also, research using stored blood units has reported increased DENV RNA and dependence on cellular translation [38], which can be interpreted as indirect evidence of viral replication. In addition to these divergences, the contribution of contaminating cells, such as residual leukocytes, especially considering similar findings in red blood cell units, which are intrinsically incapable of viral replication [7, 38, 40], cannot be ruled out. Notwithstanding the discrepancies regarding DENV platelet infection, it is feasible to assume that, whether through abortive or non‐abortive infection, DENV can contribute to inflammation and vascular dysfunction. However, the actual extent of platelet biosynthetic capacity and their involvement in the production of virions remains critical points of debate in the literature.

Dengue is a widely studied disease, but it still presents several gaps, especially regarding a broader knowledge of its clinical outcome. The World Health Organization (WHO) provides guidelines for diagnosis and severity assessment, classifying dengue into cases without warning signs, with warning signs, and severe dengue based on clinical and laboratory features [30, 41]. However, such approaches present low specificity and do not allow accurate prediction of disease progression. Thus, studies have been conducted to identify more sensitive markers capable of signaling, at an early stage, disturbances associated with dengue worsening. It is worth highlighting that disease progression depends on numerous individual factors, such as immunological, genetic, environmental, and microbiota‐associated factors [42, 43, 44].

Given the functional relevance of platelets in DENV infection and their close association with clinical outcome and poor prognosis, a better understanding of the mechanisms underlying their activation, dysfunction, and interaction with the immune system is of general relevance. Therefore, this narrative review aims to identify and discuss key platelet‐derived biomarkers involved in dengue pathophysiology. To this end, scientific articles were searched in public databases, such as PubMed, and the studies were selected based on their relevance to platelet activation, immune responses, and disease severity. Emphasis was placed on experimental evidence highlighting the immunological functions of these biomarkers, their association with clinical outcomes, their potential as prognostic markers, and their relevance as possible therapeutic targets.

## 2 Immune Response and the Role of Platelets in Dengue

The immune response against DENV typically begins with the activation of innate immunity through the recognition of pathogen‐associated molecular patterns (PAMPs) by pattern recognition receptors (PRRs) such as Toll‐like 3 (TLR3) and TLR7, and cytosolic receptors such as retinoic acid‐inducible gene I (RIG‐I) and melanoma differentiation‐associated protein 5 (MDA5), expressed primarily by immune cells [45, 46, 47]. This recognition leads to the production of type I interferons (IFN‐α/β), which promote an intracellular antiviral state characterized by the induction of interferon‐stimulated genes (ISGs) that inhibit viral replication, enhance viral RNA degradation, and limit protein synthesis, primarily via activation of the JAK–STAT and IRF3/IRF7 pathways [48, 49, 50, 51]. Mast cells and neutrophils participate in the tissue inflammatory response, releasing vasoactive mediators such as histamine, tryptase, and VEGF, which are associated with clinical manifestations such as skin rashes and vascular leakage [24, 52]. In addition, DENV nonstructural proteins (NS1, NS2A/B, NS3, NS4A/B, and NS5) interfere with key interferon pathways, including PRR‐dependent signaling and the JAK–STAT pathway, thus impairing IRF3/IRF7 activation and downstream interferon‐dependent gene expression, thereby promoting viral immune evasion [53, 54, 55]. The combination of these activation and evasion mechanisms contributes to the clinical variability of dengue, which may range from mild to severe cases such as DHF and DSS [56, 57]. Aside from the role of these molecules, there are membrane receptors that actively participate in the virus‐host interaction; notably, C‐type lectins (CD209) and CLEC5A induce the binding and activation of immune cells [58, 59]. Furthermore, phosphatidylserine receptors (TIM‐1/AXL) facilitate viral entry [60, 61] and TLR4, which is activated by secreted DENV NS1 protein, contributes to increased inflammation and endothelial dysfunction [56].

The adaptive immune response also plays a central role in defense against DENV. Dendritic cells and macrophages act as antigen‐presenting cells, activating CD4^+^ and CD8^+^ T lymphocytes, which are responsible for cytokine production and the destruction of infected cells [62, 63]. Th1 lymphocytes promote inflammation through IFN‐γ and TNF‐α/β, whereas B cells produce neutralizing antibodies that recognize structural viral proteins such as E and prM [64, 65]. However, during secondary DENV infections, preexisting non‐neutralizing or sub‐neutralizing antibodies can lead to ADE, facilitating viral entry into Fcγ receptor‐bearing cells. This process leads to increased viral replication and higher viral loads, which in turn contribute to dysregulation of the antiviral immune response and is often associated with poor prognosis [66, 67].

In addition, other innate immune pathways, such as the complement system, autophagy, and iRNA, also contribute to viral control but may be subverted by DENV [68, 69, 70]. In this context, immune recognition mechanisms in platelets are not different. Although platelets are generally known for their participation in hemostasis and clot formation, they have emerged as important cells in the immune response, especially in viral infections such as dengue. Platelets express PRRs, including TLR1, TLR4, TLR6, TLR7, and TLR9, which can recognize viral components besides triggering pro‐inflammatory signaling cascades [71, 72, 73, 74, 75, 76]. Platelet activation induces the release of pro‐inflammatory cytokines and chemokines such as CXCL4 (PF4), RANTES (CCL5), IL‐6, CXCL8, and TNF‐α, which are stored in α‐granules and contribute to the modulation of innate immunity and the recruitment of cells such as monocytes and neutrophils [77, 78, 79, 80, 81]. Furthermore, a systemic increase in type I interferons induces the expression of interferon‐stimulated genes (ISGs) in circulating platelets and megakaryocytes (MKs), particularly IFITM3, in dengue and influenza infections. Megakaryocytes infected with DENV secrete IFNα and IFNβ, robustly inducing the expression of the antiviral gene IFITM3 in MKs and platelets. IFITM3 induction confers intrinsic antiviral immunity, restricting dengue infection in MKs and limiting viral dissemination to adjacent hematopoietic stem cells in the bone marrow niche. Thus, suggesting a noncanonical role for platelets and megakaryocytes as active participants in IFN‐induced antiviral immunity [82]. In DENV infection, platelet–leukocyte interactions, mediated by P‐selectin and CD40L, have been shown to contribute to the formation of platelet aggregates besides amplifying the inflammatory response, suggesting that their involvement may intensify vascular injury and endothelial permeability [83, 84, 85].

Aside from the immunomodulatory features of platelets during DENV infection, they are also targeted by mechanisms of cell destruction in the pathophysiology of this viral infection [31]. Moreover, excessive platelet stimulation promotes phosphatidylserine exposure and the production of pro‐inflammatory molecules which are contributing players in platelet consumption and poor disease outcome [86]. In other words, platelet activation through PRRs leads to the synthesis of inflammatory mediators and, when excessive, drives cellular destruction, thus resulting in vascular leakage and thrombocytopenia observed in more severe cases of the disease. In this regard, recent research indicates that platelet biomarkers—such as P‐selectin (CD62P), PF4, RANTES, and platelet‐derived extracellular vesicles—may provide additional support in patient risk stratification and prognostic assessment [84, 87, 88, 89]. Considering the important functional features of platelets in DENV infection, we describe during the following sections, the classic biomarkers that present robust clinical and experimental evidence, followed by non‐classic/promising biomarkers.

## 3 Classic Platelet Dysfunction and Markers During Dengue Infection

As highlighted earlier, platelets play an important role in DENV pathophysiology, especially in the more severe forms of disease. The hyperactivation and dysfunction of these cells throughout the infection compromise vascular homeostasis and contribute to clinical complications. Previous studies have shown that during DENV infection, there is an increase in platelet activation and differential expression of cell surface and soluble markers, such as P‐selectin (CD62P), CD63, integrin αIIbβ3 (CD41/CD61), CD40L, and cytokines. The association among these markers with the progression of disease severity has been a constant subject of investigation [27, 36, 83, 88, 90, 91, 92, 93]. For this reason, knowledge and understanding of platelet activity, as well as the identification of specific markers, may be useful to assess dengue prognosis and/or to be explored as potential therapeutic targets.

### 3.1 CD62P

**FIGURE 1:**
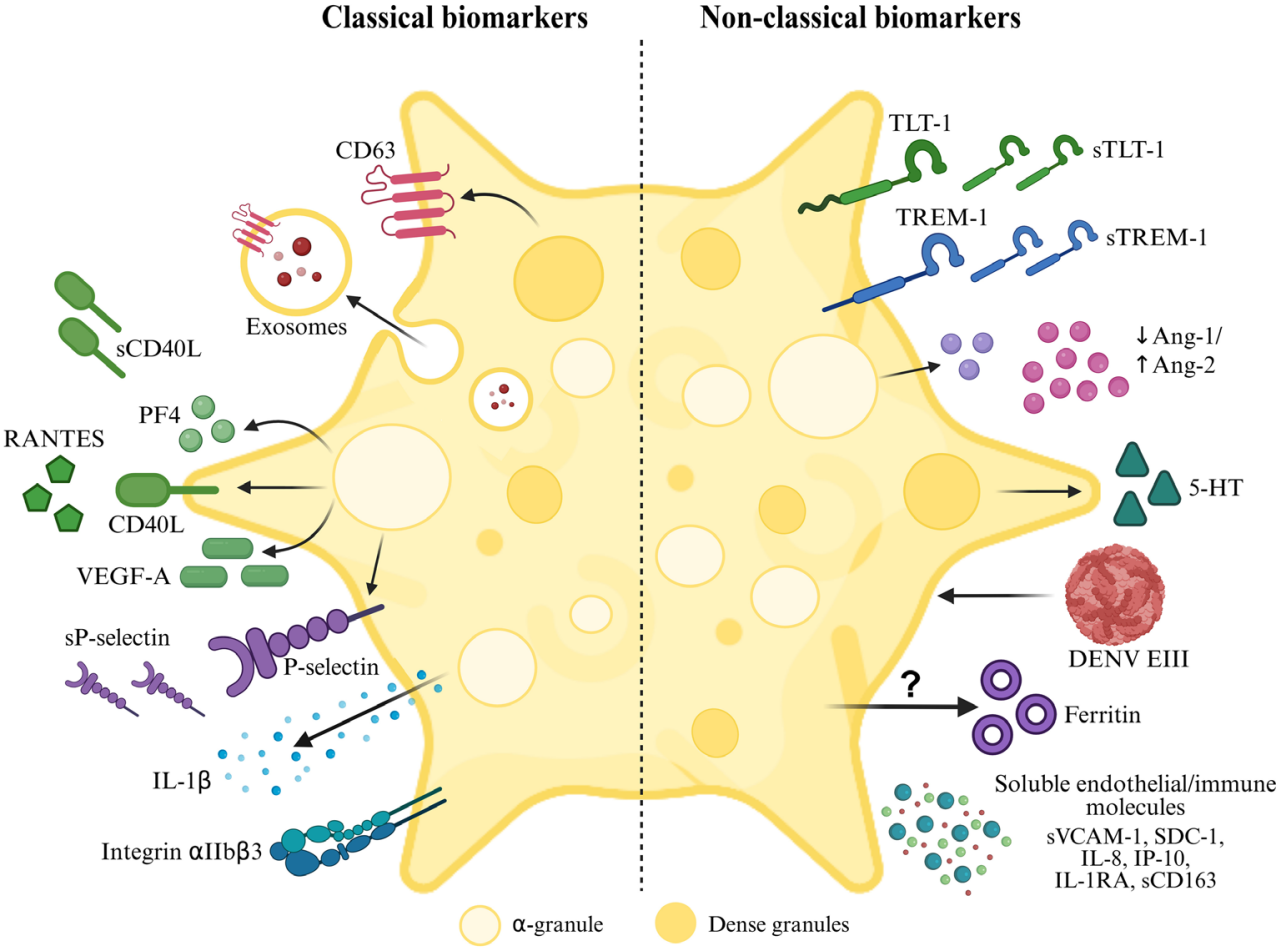
Mechanisms involved in DENV‐induced platelet activation and associated biomarkers. DENV recognition by membrane and cytoplasmic receptors in platelets, including Toll‐like receptors (TLRs) and NOD‐like receptors (NLRs), triggers platelet activation, with α‐ and dense granule release as key features. This process leads to the secretion of multiple mediators involved in leukocyte recruitment and endothelial activation, such as IL‐1β, CXCL4, CCL5, VEGF, IL‐8, IP‐10, IL‐1RA, serotonin (5‐HT), and sVCAM‐1. The α‐granule release also promotes the surface expression of molecules including P‐selectin and CD40L, which mediate interactions with leukocytes and endothelial cells, contributing to inflammation and vascular dysfunction. Additional receptors, such as TLT‐1 and TREM‐1, may also participate in DENV recognition and downstream inflammatory signaling, although their activation mechanisms remain incompletely understood. sCD40L contributes to platelet, leukocyte, and endothelial cell activation through autocrine and paracrine signaling. The functional roles of soluble TLT‐1 and TREM‐1 remain less well defined. Other soluble mediators, including 5‐HT, VEGF, and angiopoietins (Ang‐1 and Ang‐2), are associated with vascular dysfunction and have been explored as potential biomarkers. Activated platelets also release microparticles (MP) and exosomes (EX), which carry bioactive molecules capable of modulating vascular responses. In addition, DENV infection induces reactive oxygen species (ROS) production and mitochondrial dysfunction, contributing to endothelial damage, platelet apoptosis, and thrombocytopenia.

**FIGURE 2:**
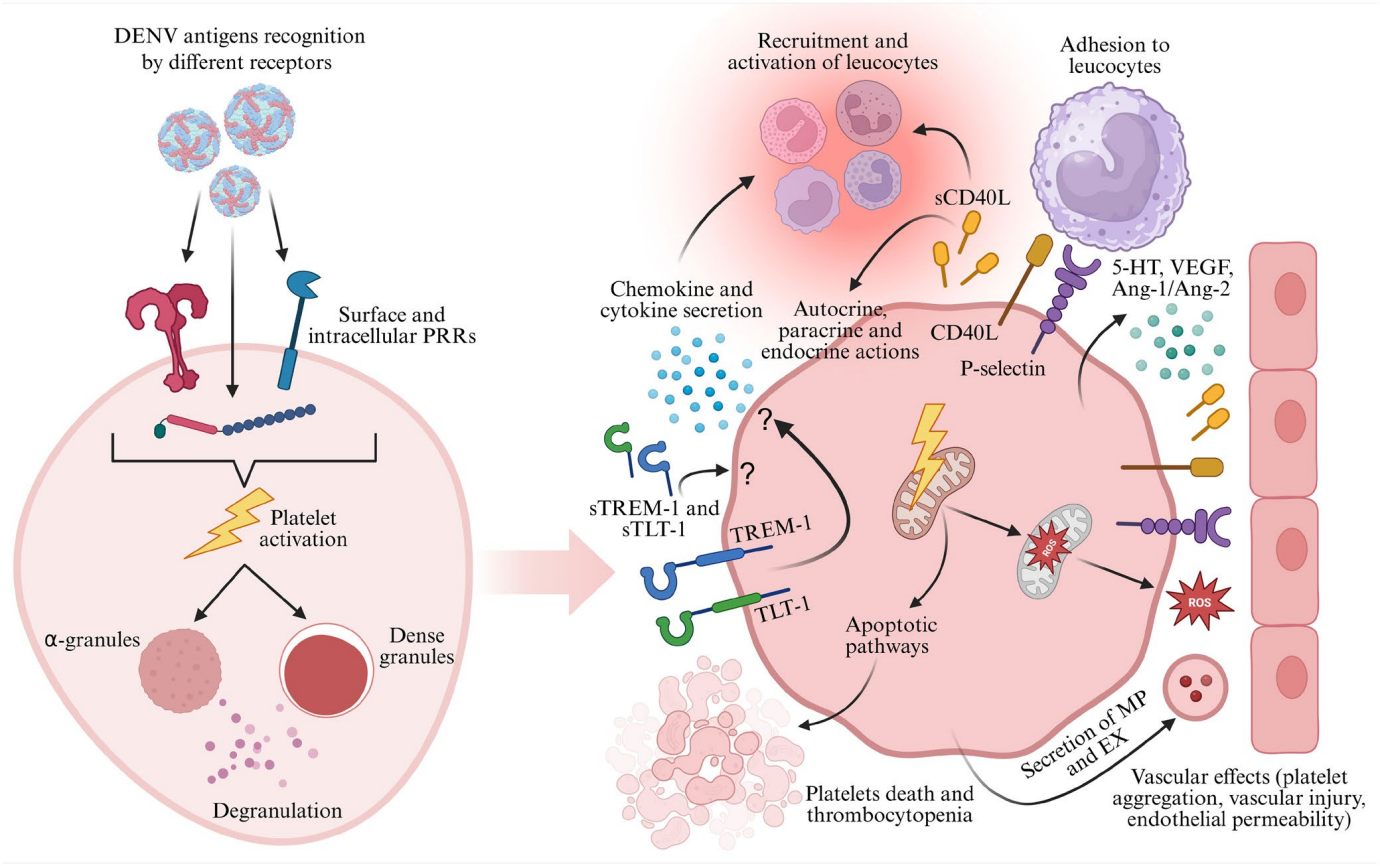
Biomarkers for platelet activation in the context of dengue virus (DENV) infection. Classical biomarkers for platelet activation include: α‐granule‐derived PF4 (CXCL4), RANTES (CCL5), VEGF‐A, P‐selectin (membrane‐associated and soluble [sP‐selectin] forms) and CD40 ligand (CD40L, membrane‐associated and soluble [sCD40L] forms); integrin αIIbβ3 (CD41/CD61), IL‐1β cytokine; dense granule‐derived CD63 and exosomes/microparticles (platelet‐derived microvesicles), which can also contain CD63 and other factors, including IL‐1β. Regarding non‐classical/potential biomarkers: the membrane‐associated immune receptors TLT‐1 and TREM‐1, besides their soluble forms, sTLT‐1 and sTREM‐1; decreased levels of the platelet‐derived angiopoietin‐1 (Ang‐1) concomitant to increased levels of angiopoietin‐2 (Ang‐2); dense granule‐derived serotonin (5‐HT); domain III of DENV envelope (E) protein; serum ferritin (from different origins, possibly from the platelet itself); different soluble molecules from endothelial and immune cells, such as IL‐8, IP‐10, interleukin 1 receptor antagonist (IL‐1RA), SDC‐1, soluble VCAM‐1 (sVCAM‐1), SDC‐1, and CD163 (sCD163).

CD62P/P‐selectin is a key marker of platelet activation and mediates the adhesion of platelets to leukocytes and the vascular endothelium [94] (Table 1). This molecule is stored in α‐granules of platelets and in endothelial cells (Figures 1 and 2). Upon cellular activation, granule contents are released, and membrane‐associated molecules are translocated to the cell surface [94] (Figure 2). Despite this, there are controversies regarding its usefulness as a severity marker in DENV infection.

**TABLE 1:** Biomarker panel in dengue fever: classical and non‐classical/emerging candidates.

	Biomarker	Type/Origin	Main clinical finding	Clinical and pathophysiological implication	References
Classic biomarkers	P‐SELECTIN (CD62‐sCD62P)	Membrane protein/α‐granules	↑ Expression in activated platelets; serum levels vary according to disease phase	Indicator of platelet activation and platelet‐leukocyte aggregates formation	[88, 90, 91]
	PF4 (CXCL4)/RANTES (CCL5)	Chemokine/α‐granules	↓ Intraplatelet content; increased circulating levels	Alpha granule release; modulate inflammation and cell recruitment	[36, 88]
	CD40L (soluble and membrane)	Transmembrane glycoprotein/α‐granules	Changes in platelet expression during dengue	Immunomodulatory role; possible biomarker of severity	[90, 92, 95]
	IL‐1β (platelets and MPs)	Inflammatory cytokine	↑ In platelets and microparticles; correlates with increased vascular permeability	Key pro‐inflammatory signal; possible target (NLRP3 inflammasome)	[83]
	VEGF‐A	Growth factor/α‐granules	↑ Serum levels in severe dengue	Pro‐vascular permeability mediator	[27, 91]
	Integrin αIIbβ3 (CD41/CD61)	Adhesion/membrane receptor	↓ Expression in patient platelets	Functional defect and may contribute to bleeding risk	[87, 93]
	CD63	Tetraspanins/dense granules and exosomes	↓ Exposure in activated platelets in some cohorts and functional defect in platelets	Suggests functional secretory defect	[87, 93]
	AST/ALT	Bloodstream	↑ Levels of AST at the onset of the disease	Lower lymphocyte counts and subsequent plasma leakage	[96]
	Platelet extracelular vesicles	Extracellular vesicles	↑ Frequency in severe dengue	Contribute to systemic inflammation and monocyte activation	[83, 87, 97]
Non‐classical/emerging biomarkers	TLT‐1 (TREM‐like transcript‐1)	Platelet receptor/α‐granules	Little studied in dengue; expressed in activated platelets	Hemostatic and inflammatory regulation (initial data)	[98, 99, 100, 101]
	TREM‐1/sTREM‐1	Immune receptor/platelets and leukocytes	↑ Levels in patient plasma	Marker of innate inflammation and permeability	[102, 103, 104, 105, 106]
	Angiopoietin‐1	Endothelial factors	↓ Plasma levels in DHF/DSS; positive correlation with platelets and albumin	Loss of the protective role of endothelial stability; associated with plasma leakage	[107]
	Angiopoietin‐2		↑ Levels in DHF/DSS; higher Ang‐2/Ang‐1 ratio in DSS	Marker of endothelial activation; promotes vascular permeability	
	Serotonin (5‐HT)	Biogenic amine/dense granules	Increased platelet serotonin levels. Altered in metabolomics of critically ill patients	May contribute to regulation of permeability and symptoms	[93, 108]
	Domain III of the E protein (EIII)	Viral protein/PAMP	Induces platelet activation and death, and is an experimental marker of severity	Induction of abnormal platelet activation and primarily necrotic cell death by pyroptosis	[109]
	Ferritin	Acute phase protein	Systemic marker of inflammation and severity, correlates with thrombocytopenia	Hyperferritinemia is a characteristic sign of macrophage activation syndrome (MAS)	[110]
	sVCAM‐1, SDC‐1, IL‐8, IP‐10, IL‐1RA, sCD163	Endothelial/inflammatory molecules	Combined markers in multimarker panels as early predictors of severe dengue		[111]

On one hand, a study showed an increase in the expression of CD62P and a subsequent increase in platelet activation in all patients with dengue, regardless of disease severity [90]. The study included 71 participants with different clinical forms of DENV infection and 19 healthy subjects. The analysis only compared individuals with dengue fever versus healthy individuals, without considering the clinical form and without mentioning the period of sample collection after the onset of the disease [90]. On the other hand, another study compared 69 samples collected up to 10 days after the onset of the disease and compared them to 28 samples from healthy individuals. The authors demonstrated that surface expression of CD62P was higher in patients with dengue with warning signs and in severe dengue compared to patients with dengue without warning signs. This suggests that disease severity was linked to platelet activation [87]. In accordance, it has been demonstrated that the expression of CD62P on the platelet surface is increased in thrombocytopenic patients when compared to nonthrombocytopenic individuals [112].

Platelets are a major source of soluble CD62P (sCD62P) [91]. A study showed that there was no significant difference in plasma levels of sCD62P between patients with and without plasma leakage [93]. Furthermore, similar plasma values of sCD62P were found in all dengue patients regardless of disease severity, and between dengue patients and healthy donors. However, the authors emphasize that platelet count may interfere with plasma sCD62P content and therefore suggest that the ratio of sCD62P to platelet count may work as an alternative approach to predict clinical stage of the disease. This was proposed based on the observation that this ratio was higher in patients with dengue with warning signs and severe dengue when compared to healthy controls. A reduction in sCD62P/platelet count ratio was also observed in cases of mild dengue compared to dengue with warning signs and severe dengue [91]. The CD62P and P‐selectin glycoprotein ligand‐1 (PSGL‐1) participate in a biologically important thrombo‐inflammatory axis in dengue infection. The CD62P actively participates in the mobilization of alpha granules and platelet activation, while PSGL‐1, in leukocytes, contributes to leukocyte adhesion [84, 113]. The accumulation of these complexes results in the formation of platelet‐leukocyte aggregates capable of increasing cytokine production and vascular dysfunction. Thus, both CD62P and PSGL‐1 can be considered mechanistic biomarkers of activation, while the platelet‐leukocyte aggregate can be considered functional and effector biomarkers in disease progression [113, 114]. These findings reinforce the importance of exploring CD62P as a biomarker for dengue progression.

### 3.2 CD63, CD41, and CD61

Analysis of platelet‐derived exosomes, a type of extracellular vesicle, reveals CD63 and integrin αIIbβ3 as indicators of vascular barrier integrity dysfunction and platelet dysfunction, respectively (Table 1) [87]. CD63 is a membrane protein of platelet dense granules, lysosomes, and exosomes and is described as another marker of platelet activation (Figure 2). The protein has also been used as a marker to indicate dense granule secretion, as is observed on the lysosomal surface of platelets [115], which represents a major contributor to maintaining vascular integrity. In this context, CD63 represents a functional marker of platelet granule/lysosomal mobilization and can be considered a biomarker in dengue fever [116, 117]. These platelet alterations go beyond the well‐known thrombocytopenia in dengue fever, as they involve abnormal activation, dysregulated granule secretion, and loss of function [93]. The CD63 expression reflects not only platelet activation but also an impairment in platelet function itself. This is important because, in addition to this dysregulation being associated with plasma leakage, it also suggests that the preservation of platelet granule function may be directly linked to the maintenance of vascular integrity [93].

Integrin αIIbβ3 is a heterodimer formed by CD41 (integrin αIIb) and CD61 (integrin β3) observed in the platelet membrane. This complex is the main fibrinogen receptor in platelets and is essential for platelet aggregation [118]. In exosome investigations, the combination of these markers is used to specifically identify platelet‐derived exosomes, as CD63 confirms the vesicle as an exosome and CD41/61 indicates its platelet origin [81]. In the context of DENV infection, this combination is employed to characterize platelet activation and function [93]. Decreased expression of CD63 and CD41/CD61 in platelets from dengue patients in the febrile and critical phase has been implicated in the abrogation of adhesion/aggregation when stimulated ex vivo, and patients with reduced expression of these markers showed a higher risk of plasma leakage [93] (Table 1). Furthermore, these patients also exhibited a reduction in platelet interactions with monocytes and neutrophils. These interactions play an essential role in modulating the inflammatory response and preserving the integrity of the endothelial barrier [93]. Platelet‐derived exosomes (PLT‐EXOs) from dengue patients express higher levels of CD63 proteins compared to healthy individuals. These extracellular vesicles play a role in disease severity by promoting increased vascular permeability [87, 97]. A significant reduction in integrin αIIbβ3 expression was observed on the surface of platelets from patients with DENV infection with platelet count less than 50 000/mL. Although this did not correlate with disease severity, the researchers argued that the loss of integrin αIIbβ3 expression may impair platelet aggregation and thrombus formation, ultimately contributing to hemorrhagic manifestations during the course of the disease [90]. Additionally, it should be noted that during DENV infection, platelet activation increases the exposure of phosphatidylserine (PS) on the outer surface of the platelet membrane. This PS exposure plays an important procoagulant role but also makes platelets susceptible to phagocytosis, contributing to their destruction and depletion in circulation [86]. Since integrin αIIbβ3 is associated with the procoagulant platelet profile [93, 118], its reduced expression may reflect the preferential elimination of PS‐exposing platelets.

### 3.3 CD40L

CD40L (CD154) is a type II transmembrane protein observed in α‐granules of platelets and works as a ligand for the CD40 receptor. Upon activation by stimuli such as thrombin, collagen, and ADP, platelets translocate CD40L to their surface or release its soluble form (sCD40L) following cleavage of the membrane‐bound protein (Figure 1). The sCD40L exhibits pro‐inflammatory activity, mediates platelet‐leukocyte interactions [78, 119, 120, 121], and contributes to vascular inflammation in atherosclerosis and thrombotic disorders [122]. Surface expression of CD40L on platelets can interact with CD40 on endothelial cells, monocytes and macrophages (Table 1, Figure 1) [119]. During interaction with endothelial cells, it induces the expression of adhesion molecules (ICAM‐1 and VCAM‐1), as well as induces the production of chemokines and cytokines that promote leukocyte recruitment [78]. Furthermore, CD40L exerts autocrine and paracrine signaling in platelets, as both sCD40L and membrane‐bound CD40L can activate other platelets or enhance their activation via receptors such as CD40 or integrin αIIbβ3 [123, 124].

Platelet CD40L surface expression, as well as the serum concentrations of sCD40L, appears to be modulated by the number of circulating platelets and the stage of DENV infection. A study observed a trend towards increased CD40L expression in platelets from dengue patients according to clinical severity. However, the authors did not specify the disease stage (febrile, critical, and recovery phases) at which the analyses were conducted, which may represent an important confounding factor influencing CD40L dynamics [90]. It is worth noting that human platelets stimulated in vitro with DENV, rapidly (within 2 h), increased the secretion of sCD40L, but these levels declined after 4 h, although they remained significantly higher than the uninfected group [95]. Despite that, clinical observations showed reduced circulating sCD40L levels in dengue patients with vascular leakage [92]. This apparent divergence can be reconciled by the strong positive correlation between sCD40L levels and platelet counts, suggesting that the reduction in sCD40L may be related to platelet depletion. While individual platelets may secrete more sCD40L upon viral stimulation, the systemic reduction in platelet counts during severe dengue ultimately leads to lower overall levels [92]. Taken together, these data support an association between CD40L and dengue severity, although its relationship with vascular permeability appears to be indirect and largely influenced by platelet counts [92]. Thus, CD40L may have potential as a biomarker in dengue, but its interpretation requires careful consideration of platelet dynamics.

### 3.4 Histone H2A

DENV infection can induce the formation of neutrophil extracellular traps (NETs), leading to the release of histones into the circulation, which are associated with disease severity [88, 125]. Evidence indicates that circulating histone H2A enhances platelet activation in dengue and contributes to disease pathogenesis. Elevated levels of H2A have been observed in the plasma of dengue patients, particularly in severe cases [88]. Similarly, H2A has been detected in platelets from infected patients but not in those from healthy volunteers [88]. Importantly, DENV infection does not directly induce H2A expression by platelets; rather, platelets capture free histones present in peripheral circulation. In vitro assays demonstrate that platelets from healthy individuals, when incubated with recombinant human H2A, show a significant increase in CD62P translocation to the surface [88]. This activation is partly mediated by H2A binding to TLR4, which ultimately enhances the release of pro‐inflammatory molecules such as PF4/CXCL4 [88]. Thus, H2A‐induced platelet activation may play an important role in dengue outcome, although further studies are needed to clarify its exact contribution to disease pathophysiology.

## 4 Inflammatory Markers

Data from in vitro experiments have demonstrated that higher DENV genome copy numbers in platelets are directly correlated with increased platelet activation, production of pro‐inflammatory extracellular vesicles, and accelerated phagocytosis by monocytes [86]. Platelet hyperactivation results in increased surface binding of the complement protein C3 and IgG. At the same time, platelet activation triggers the activation of caspase 9 and cyclophilin D, which are proapoptotic proteins, as well as increased phosphatidylserine surface exposure. These findings suggest that platelet activation plays a decisive role in thrombocytopenia and increased inflammation activation during dengue infection [86].

In addition to the cellular and soluble markers mentioned so far, platelet hyperactivation during DENV infection results in excessive secretion of cytokines and chemokines stored in α‐granules [27, 36, 83, 88, 91] and dense granules [93, 126]. Among the molecules secreted and stored in α‐granules, PF4/CXCL4, RANTES/CCL5 (Figure 2) [36, 88], and VEGF [27, 91] are noteworthy. Dense granules, in turn, release mediators such as ADP and serotonin [108]. The role of these mediators is discussed later in this review.

PF4 plays a key role in hemostasis and thrombosis. It also acts as an immunomodulatory molecule by inducing the chemotaxis of monocytes and neutrophils, thereby enhancing inflammatory responses [127]. Also secreted by activated platelets, RANTES (CCL5) is a chemokine involved in the recruitment of leukocytes—such as monocytes and T lymphocytes, through interaction with CCR1 and CCR5 receptors [128, 129].

Dengue patients showed decreased intraplatelet content of PF4/CXCL4 and RANTES/CCL5 when compared to healthy controls [88]. Additionally, increased plasma levels of PF4/CXCL4 were observed in dengue patients in comparison to healthy controls [88]. These results suggest that during dengue infection, in vivo platelet activation promotes the progressive release of stored PF4/CXCL4 and RANTES/CCL5, thus contributing to the reduction of their intracellular content [88]. This hypothesis was confirmed by in vitro assays, in which platelets from healthy volunteers were stimulated with DENV and exhibited increased secretion of the chemokines PF4/CXCL4 and RANTES/CCL5 stored in granule [36]. Another point of interest is that the secretion of PF4/CXCL4 by DENV‐activated platelets induces increased viral replication in monocytes (Table 1) [130].

Platelets contribute to increased vascular permeability in DENV infection through the release of interleukin‐1 beta (IL‐1β) in a NLRP3‐dependent way, which results in the production of mitochondrial reactive oxygen species (mtROS) [83]. Dengue patients with increased vascular permeability, evidenced by signs such as a 20% increase in hematocrit, hypoalbuminemia, postural hypotension, ascites, and/or oliguria, had a higher percentage of platelets containing IL‐1β and IL‐1β‐rich platelet‐derived extracellular vesicles [83].

Mitochondrial dysfunction in platelets and endothelial cells has also been associated with dengue pathogenesis [112, 131]. Basal production of mitochondrial reactive oxygen species (ROS) is higher in platelets from dengue patients. Moreover, dengue infection impairs mitochondrial function in platelets which ultimately induces the activation of apoptotic pathways with increased PS exposure [112]. This ultimately leads to thrombocytopenia [86, 112], as well as enhancing the release of vesicles rich in pro‐inflammatory [83] and procoagulant mediators [112]. Disturbances in mitochondrial membrane potential and increased ROS are also known for their role as indicators of oxidative stress in endothelial cells [112, 131]. These factors contribute to reduced platelet viability and the promotion of endothelial dysfunction, which together may worsen the vascular impairments observed in severe dengue.

## 5 Vascular Endotelial Mediators

### 5.1 Vascular Endothelial Growth Factor

Vascular endothelial growth factor (VEGF) is part of a family of signaling glycoproteins involved in the formation of blood vessels (angiogenesis) and lymphatic vessels (lymphangiogenesis) [132, 133]. Although it comprises different isoforms, VEGF‐A has been the most extensively studied so far. VEGF‐A predominantly interacts with tyrosine kinase receptors VEGFR‐1 and VEGFR‐2, triggering cellular signaling pathways that promote endothelial cell proliferation, migration, and survival (Figure 1) [133, 134]. Biologically, VEGF plays a significant role in tissue repair and in the adaptation of tissues under hypoxic conditions [133, 135]. Under pathological conditions, increased VEGF expression may contribute to tumor growth, proliferative retinopathies, macular degeneration, among other disorders. For this reason, the development of VEGF inhibitors has been encouraged, aiming to suppress excessive angiogenesis and reduce pathogenic vascular permeability [133, 136].

Elevated levels of VEGF in serum or plasma have been correlated with increase in vascular permeability in severe dengue [27]. Platelet activation stimulates the release of VEGF stored in α‐granules [27, 91]. Analysis of serum VEGF levels in 106 patients (58 with non‐severe dengue and 48 with severe dengue), compared to healthy individuals, showed that VEGF levels were lowest in controls, increased in non‐severe dengue, and were significantly higher in severe dengue [92]. By contrast, VEGF levels were higher in patients with severe dengue when compared to those with non‐severe dengue [27]. These data suggest that VEGF, due to its different profiles during dengue infection, may be used as a predictive marker for dengue progression and severity (Table 1). Additionally, elevated VEGF levels were associated with thrombocytopenia and increased hematocrit levels [24, 25, 27, 137]. Another study showed that although not statistically significant, there was a strong trend towards elevated circulating VEGF levels in patients with mild dengue compared to those with dengue with warning signs [91]. Nevertheless, only 15% of the cohort was classified as severe dengue, which may have limited the ability to detect differences across severity groups [91]. These divergent findings across studies highlight the need for a more detailed investigation of VEGF dynamics in dengue, considering the different days of illness progression as well as the distinct clinical classification.

### 5.2 Serotonin

Regarding mediators originated from platelet dense granules, serotonin is well recognized for its role in vascular permeability [93, 138]. It is not synthesized by platelets but is primarily produced by peripheral cells, such as enterochromaffin and mast cells [139]. Subsequently, these molecules are taken up, stored in platelet dense granules, and released upon platelet activation [139]. Increased intraplatelet serotonin levels have been observed in dengue patients and implicated in plasma leakage [93, 139, 140, 141]. Platelet activation by DENV induces the release of serotonin (Figure 2), further increasing platelet aggregation. The release of serotonin may play a reliable role as an indicator of disease severity. Comparative analyses between individuals with dengue fever and those with dengue hemorrhagic fever, employing metabolomic approaches and evaluating serum metabolites derived from platelets, revealed a significant reduction in serotonin concentrations in the latter group. The study concluded that serotonin, together with IFN‐γ, is a good predictor of disease severity [108]. However, the authors emphasize that for clinical application, the development of rapid and cost‐effective analytical methods for the quantification of serotonin and IFN‐γ is required, along with further validation in an independent cohort [108]. Furthermore, it was shown that serotonin released by mast cells amplifies platelet activation via the 5HT2A receptor, enhancing the platelet response to the virus, promoting platelet aggregation and splenic removal, ultimately resulting in thrombocytopenia in vivo, without a direct impact on viremia. Platelet activation and decreased platelet count seem to be correlated in dengue patients, as well as the need for competent mast cells for serotonin synthesis to induce the phenotype in murine models. Pharmacological blockade of the 5HT2A receptor demonstrated a reduction in platelet activation and thrombocytopenia associated with DENV, with no negative impact towards viral infection outcome [142]. Thus, indicating the mast cell‐serotonin‐platelet pathway as a promising therapeutic target based on host factors, with potential applicability in other hemorrhagic viral infections [142]. Moreover, serotonin levels should be assessed in other acute febrile infectious diseases as well as in patients infected with different DENV serotypes [108]. Taken together, these data support a notion that platelets act primarily as a reservoir and delivery system for serotonin, linking immune and vascular responses. Further studies evaluating serotonin levels across different disease states and DENV serotypes may help refine its specificity and applicability as a biomarker and therapeutic target in dengue.

## 6 Adenosine Diphosphate (ADP)

ADP is a purinergic agonist released by activated platelets that amplifies the thrombotic response [143]. It binds primarily to P2Y_1_ and P2Y_12_ receptors, promoting conformational changes in platelet integrins, particularly αIIbβ3, leading to granule secretion and platelet aggregation [143]. In an in vitro study, DENV NS1 alone could not induce platelet aggregation, but it was shown that NS1‐mediated activation can trigger ADP secretion, which together enhance platelet aggregation [126]. Evidence suggests that circulating DENV NS1 binds to platelet TLR4 or other molecules, inducing the release of ADP [126]. This process, in turn, increases P‐selectin expression and PS exposure on the platelet surface, promoting platelet activation and aggregation. Platelets activated by NS1 tend to adhere to the endothelium or be phagocytosed by macrophages [126]. The ADP, after vascular injury, also amplifies the activation of other platelets. These effects, together, may contribute to endothelial injury and explain the thrombocytopenia and bleeding episodes observed during DENV infection [126]. However, ADP acts in a highly localized and transient manner at sites of platelet activation, limiting its utility as a circulating diagnostic or prognostic biomarker. Therefore, its relevance in this context lies in its role as a key mediator of platelet activation rather than as a measurable biomarker of disease severity.

So far, we have shown different forms in which DENV infection disturbs platelet function, ranging from hyperactivation and dysregulated granule release to mitochondrial dysfunctions that compromise platelet survival and effectiveness in modulating the immune response. The identification of specific biomarkers may assist in the early diagnosis of disease severity and in the adoption of more effective therapeutic strategies to mitigate its clinical impacts (Table 1).

## 7 Non‐Classical Platelet Biomarkers as Potential Prognostic Markers in Dengue

So far, we have described markers that have been directly or indirectly explored in dengue severity. However, based on the central role of platelet activation, clot disturbances, and vascular disturbances observed in the disease poor outcome, the role of other molecules in this scenario cannot be ruled out. In this regard, within the Triggering Receptor Expressed on Myeloid Cells (TREM) family of immune receptors, a member known as the transcript similar to triggering receptor expressed on myeloid cells‐like transcript 1 (TLT‐1) has been suggested as a more sensitive marker of platelet activation than CD62P [98] (Table 1). The TLT‐1 is a receptor that has two main forms, the membrane form (mTLT‐1) composed of an extracellular IgV domain (with 147 amino acids), a transmembrane domain (21 amino acids) and a cytoplasmic domain (with 127 amino acids) [144], and soluble form (sTLT‐1), resulting from the cleavage of the extracellular domain, being found in the serum of humans and mice [145]. The receptor is specifically found in the α‐granules of megakaryocytes and platelets, from which it can be rapidly translocated to the surface upon platelet activation or secreted into the bloodstream in its sTLT‐1 isoform [98, 99]. In vitro and in vivo assays have been shown that TLT‐1 is translocated to the platelet surface earlier than CD62P [98].

Furthermore, sTLT‐1 has been described as a marker associated with increased platelet aggregation and adhesion to the endothelium, where it stimulates actin polymerization, adhesion, and platelet spreading [100]. These effects increase platelet‐endothelium interactions and contribute to the maintenance of hemostasis but may also be related to venous thrombosis in inflammatory conditions [100] (Table 1). Beyond its role in regulating leukocyte activation and modulating the acute inflammatory response induced by sepsis [101], TLT‐1 has also been proposed as a potential marker of platelet activation in preeclampsia [99]. Although the association between TLT‐1 and DENV infection has not yet been directly explored, it is biologically plausible to hypothesize that the virus, by inducing platelet activation and dysfunction, may also promote the release of sTLT‐1, leading to elevated serum levels. In this context, sTLT‐1 emerges as a promising candidate biomarker of dengue severity. Collectively, these findings underscore the prominent role of TLT‐1 in hemostasis [146] and support the hypothesis that it may play a role in the pathophysiology and influence the clinical outcomes of dengue, particularly in more severe cases.

Identifying novel platelet‐related biomarkers is essential to improve our understanding of the mechanisms underlying severe dengue complications. In this context, TREM‐1, which is also, but not exclusively, expressed in platelets, emerges as a promising candidate biomarker for dengue. TREM‐1 has been widely associated with the amplification of the immune response in both infectious and noninfectious conditions, including viral infections [147, 148, 149] (Table 1, Figures 1 and 2). One study associated dengue virus type 2 infection with a significant increase in TREM‐1 and CD10 expression on neutrophils [102]. The main feature of this receptor, which is the amplification of inflammation, may partly explain the severity of the disease through the increased expression of TREM‐1 [102]. Furthermore, there was a significant increase in serum levels of soluble TREM‐1 (sTREM‐1) in the first 5 days after the onset of symptoms in the blood of individuals infected with dengue compared to healthy controls [103].

On the other hand, circulating levels of sTREM‐1, evaluated in more than 244 individuals with dengue (63 without warning signs, 179 with warning signs, and 2 with severe disease), demonstrated that patients with elevated sTREM‐1 levels, compared with healthy controls, were 3.8 times more likely to develop hemoconcentration, a condition characterized by increased red blood cell concentration in the bloodstream. This process directly contributes to plasma extravasation. Furthermore, its increase was linked to an increase in other markers of endothelial activation, such as Ang2, soluble tyrosine kinase‐1 (sFlt‐1), and vascular adhesion molecule (sVCAM‐1) [150]. These findings suggest that components of the TREM family may be extremely sensitive in predicting worsening outcomes in dengue infection.

It is important to emphasize that, like TLT‐1, TREM‐1 is stored in platelet α‐granules. Its inhibition has been shown to reduce collagen‐ and thrombin‐induced platelet activation and aggregation, as well as thrombus formation, highlighting its role in thrombo‐inflammatory and hemostatic processes, including conditions such as pulmonary embolism [144, 151, 152]. These findings provide a mechanistic basis to explore the involvement of TREM‐1 in dengue, a disease characterized by platelet dysfunction and coagulopathy. In this context, TREM‐1 may contribute to the dysregulated hemostatic response observed in severe DENV infection, and its modulation could represent a potential target for future investigation. In addition, it is well known that NS1 activates platelets via TLR4, inducing degranulation (increased CD62P), aggregation, and greater platelet clearance [126, 153, 154]. It is noteworthy that TREM‐1 has already been shown to act synergistically with TLR4 [104, 105, 106]. This suggests that the activation of both TREM‐1 and TLR4, known to be pro‐inflammatory, may represent key contributors to the exacerbated immune response observed in dengue besides causing a marked increase in disease severity. Therefore, we suggest that studies focusing on the mechanisms of TLT‐1 and TREM‐1 in dengue infection could help to elucidate possible roles of these receptors in platelets and disease pathophysiology. Receptors of the TREM family are already being explored as therapeutic targets in viral infections, such as COVID‐19 [155, 156] (Table 2).

**TABLE 2:** Panel of biomarkers in dengue fever that can be explored as therapeutic targets and next steps for validation.

Therapeutic target	Biomarker	Evidence in dengue	Next steps for validation	References
Endothelial stabilization—TIE2 AXIS	Ang‐1 ↓ and Ang‐2 ↑	Human (pediatric) cohort and association with plasma extravasation (albumin and PEI)	Longitudinal studies by disease phase, TIE2 axis modulation assays in models and validation as a prognostic panel	[107]
Platelet activation blockade	↑ Surface P‐selectin (CD62P) and ↑ sCD62P/platelets	Humans (observational/ex vivo study) and correlation with gravity	Standardization of collection and processing time, in addition to establishing cut‐offs and sCD62P/platelet index as a requestor	[88, 90, 91]
Interfere with platelet‐leukocyte aggregates (PLAs)	P‐selectin/PSGL‐1‐mediated PLAs	Humans (observational) and functional ex vivo	Test P‐selectin/PSGL‐1 blockade in dengue models and ex vivo, in addition to integrating PLAs into risk panels	[84]
Target NLRP3/IL‐1	↑ Intraplatelet and extracellular vesicle IL‐1β	Humans (ex vivo) with association with leakage; cellular mechanisms described	Proof of concept with NLRP3/anti‐IL‐1 inhibitors in models and kinetics by clinical phase	[83]
Virus activation blocking	NS1–TLR4 in platelets (degranulation, ↑ CD62P and aggregation)	In vitro/human ex vivo	TLR4/anti‐NS1 antagonist trials in models and therapeutic window mapping	[126, 153, 154]
Platelet immune co‐stimulation	CD40L (membrane/soluble sCD40L)	Observational study in humans with dengue (increase and subsequent reduction associated with thrombocytopenia at different stages)	Studies with serial samples and integration with endothelial markers	[90, 92, 95]
Preservation of platelet granule function	↓ CD63 (granule secretion) in some cohorts and functional defect in platelets	Humans (ex vivo)	Standardized functional tests and correlation with clinical outcomes	[87, 93]
Maintenance of adequate hemostatic adhesion/agonism	↓ Integrin αIIbβ3 (CD41/CD61)	Humans (observational) and suggested functional impact	Relate loss of αIIbβ3 with bleeding and transfusion needs and verify reversibility	[87, 93]
Modulation of α‐granule chemokines	PF4/CXCL4 and RANTES/CCL5: ↓ intraplatelet and elevated plasma content	Humans (ex vivo/observational)	Monitoring in different phases (febrile, critical, recovery) and evaluating selective neutralization in experimental models	[36, 88]
Attenuate pro‐permeability signaling	↑ Serum VEGF in severe dengue and relationship with thrombocytopenia/hematocrit	Observational human studies	Evaluate and validate as part of a panel (with Ang‐2/SDC‐1/IP‐10), in addition to exploring anti‐VEGF in experimental models	[27, 91]
Regulation of dense granule mediators	Altered (↑) platelet serotonin and association with severity through metabolomics	Humans (clinical metabolomics)	Studies that explore the mechanisms relating serotonin to permeability and verify its early prognostic utility	[93, 108, 142]
Reduction of pro‐inflammatory vesiculation	↑ Platelet microvesicles/exosomes and association with plasma leakage	Human studies (observational/ex vivo functional)	Standardize phenotype (CD41a, CD63); test release blockade/effects on endothelium	[83, 87, 97]
Explore emerging platelet receptors	TLT‐1 (TREM‐like transcript‐1)	Translational evidence (thrombosis/sepsis) and plausibility in dengue	Expression studies (surface/sTLT‐1) by phase; function and outcomes	[98, 99, 100, 101]
Reduction of myeloid‐endothelial cell signaling	↑ TREM‐1/sTREM‐1; association with hemoconcentration and endothelial activation	Human studies (observational adults/children); synergy with TLR4	Serial cohorts; evaluation of receptors as early predictors; exploring TREM‐1 inhibition in experimental models	[102, 103, 104, 105, 106, 150]

To date, no studies have demonstrated the therapeutic modulation of these markers in DENV infection. Nevertheless, the TREM receptor family, particularly TREM‐1, emerges as a promising candidate not only as a predictor of disease severity but also as a potential therapeutic target. Supporting this possibility, a 2020 transcriptomic study in enterovirus A71 infection showed that pharmacological inhibition of the TREM‐1 pathway using LP17 significantly reduced the expression of pro‐inflammatory genes and decreased viral replication [157]. Additionally, another study demonstrated that infection of THP‐1 cells with rotavirus induced significant production of IL‐1β, which was drastically reduced following inhibition of TREM‐1 with the compound VJDT (TREM‐1 inhibitor that suppresses receptor signaling), indicating that this receptor acts as an amplifier of the inflammatory response mediated by this cytokine. Although TNF‐α did not show a significant increase after infection, its production was also reduced upon TREM‐1 inhibition, reinforcing the modulatory role of this receptor in the immune response. Furthermore, in MA104 epithelial cells, inhibition of TREM‐1, using either VJDT or the LR12 peptide (a synthetic TREM‐1 inhibitory peptide that blocks receptor signaling), resulted in increased cell viability and reduced virus‐induced cytopathic effects, indicating that TREM‐1 contributes not only to inflammation but also to the progression of viral infection and the associated cellular damage. Although these findings were not obtained in DENV infection, they suggest that modulation of TREM‐1 may represent a promising therapeutic avenue to be explored in dengue and other viral diseases [158].

Low platelet count remains the main warning sign for dengue worsening [159, 160]. Infected individuals, before progressing to severe dengue and manifesting the most severe symptoms, often present severe thrombocytopenia. The DENV E protein domain III (EIII) represents an example of a virus‐derived biomarker associated with dengue severity (Table 1, Figure 2). The EIII is a protein from the DENV envelope, containing an Ig‐like domain, and is involved in viral binding to host cells [161]. Although not platelet‐derived, its relevance in this context lies in its direct effects on platelet biology. A 2021 study demonstrated that EIII can induce both platelet activation and cell death, leading to significantly higher levels of platelet death compared to NS1 [109]. In addition, EIII was shown to trigger the coagulation cascade and promote thrombocytopenia in mice through direct binding to platelets [109]. Therefore, these findings may at least partly support the notion of EIII as a putative biomarker indirectly linked to platelet dysfunction and dengue severity.

Another important predictive marker of dengue severity is serum levels of aspartate aminotransferase (AST) and alanine aminotransferase (ALT). These are liver enzymes released into the bloodstream in response to tissue damage caused by DENV (Table 1). An increase in the concentration of liver enzymes is commonly considered a marker of liver dysfunction, inflammation, and a predictor of severity [162, 163]. However, alterations in serum levels of these aminotransferases may reflect not only hepatocellular injury but also mitochondrial bioenergetic disturbances and disruptions in intermediary metabolism [164]. ALT catalyzes the reversible conversion between alanine and pyruvate, thereby contributing to the production of acetyl‐CoA or oxaloacetate. AST catalyzes the conversion between aspartate and oxaloacetate, establishing a direct link with tricaboroxylic acid (TCA) [164]. Although elevations in serum ALT and AST do not directly influence the TCA cycle, they reflect alterations in intracellular metabolic fluxes. As key enzymes linking amino acid metabolism to TCA intermediates such as pyruvate and oxaloacetate, their dysregulation indicates impaired anaplerotic input and mitochondrial dysfunction, which may ultimately contribute to oxidative stress and increased disease severity [164, 165].

In a study conducted in 122 non‐pediatric patients with laboratory‐confirmed dengue, elevated AST levels were associated with leukopenia, reflecting systemic involvement beyond isolated hepatic injury [96]. Moreover, infection with DENV induces mitochondrial dysfunction in HepG2 cells, characterized by reduced mitochondrial membrane potential, decreased ATP levels, and metabolic stress preceding cell death [166]. In parallel, it promotes metabolic reprogramming with increased glycolysis, enhanced glucose uptake, and dependence on this pathway for viral replication [167]. The observed mitochondrial dysfunction, including reduced membrane potential and ATP depletion, together with increased reliance on glycolysis, suggests an impaired or functionally truncated TCA cycle during dengue virus infection [166, 167].

As the liver is the primary site of thrombopoietin synthesis, hepatic dysfunction may impair thrombopoietin production, a mechanism well established in liver disease and closely linked to reduced platelet production [168, 169]. In dengue, increased aminotransferase levels have been consistently associated with disease severity and thrombocytopenia [170, 171], suggesting that liver dysfunction may contribute, at least in part, to impaired platelet homeostasis. Taken together, these findings support the use of serum AST and ALT levels as accessible biomarkers associated with disease severity and hematological alterations in dengue.

To improve the sensitivity of biomarker panels for predicting dengue severity, previous studies have evaluated combinations of markers reflecting distinct, but interconnected biological processes, including immune activation, monocyte/macrophage response, endothelial dysfunction, and systemic inflammation [111]. Importantly, although these biomarkers are not primarily platelet‐derived, they are functionally linked to pathways that modulate platelet activation, platelet‐endothelial interaction, and vascular permeability in dengue.

In this context, a case–control study including 281 patients with moderate or severe dengue and 556 patients without warning signs used a panel comprising interleukin‐8 (IL‐8) and interferon gamma‐induced protein‐10 (IP‐10), associated with immune activation; IL‐1 receptor antagonist (IL‐1RA), soluble triggering receptor expressed on myeloid cells‐1 (sTREM‐1), and soluble CD163 (sCD163), reflecting monocyte and macrophage activation; angiopoietin‐2 (Ang‐2) and syndecan‐1 (SDC‐1), related to endothelial activation and glycocalyx disruption; and ferritin and C‐reactive protein as markers of systemic inflammation [111]. The authors demonstrated that the combination of IL‐1RA, Ang‐2, IL‐8, ferritin, IP‐10, and SDC‐1 predicted disease severity with higher sensitivity in children, whereas SDC‐1, IL‐8, ferritin, sTREM‐1, IL‐1RA, IP‐10, and sCD163 were more effective in adults. Notably, some of these markers—particularly Ang‐2 and SDC‐1—are directly associated with endothelial activation and glycocalyx degradation, which are closely linked to platelet adhesion, activation, and the regulation of vascular integrity [107]. The imbalance between angiopoietin‐1 (Ang‐1), a platelet‐derived factor, and Ang‐2, an endothelium‐derived molecule, further highlights the interaction between platelets and endothelial dysfunction in dengue. Reduced levels of Ang‐1, along with platelet count and CD62P expression, suggest that thrombocytopenia and platelet activation impair the stabilizing signaling of the tyrosine kinase axis with immunoglobulin‐like domains and EGF 2 (TIE2), thus contributing to increased vascular permeability and plasma extravasation [107, 111].

Ferritin has also been proposed as a highly sensitive marker of dengue severity. Although not platelet‐derived, hyperferritinemia reflects intense systemic inflammation, which is known to promote platelet activation, consumption, and coagulopathy [110]. Elevated ferritin levels have been associated with severe thrombocytopenia, reaching over 93% sensitivity and a predictive value of 98% in a cohort of 200 dengue patients [110, 172] (Figure 2).

Increased levels of soluble vascular cell adhesion molecule‐1 (sVCAM‐1) and interferon‐gamma (IFN‐γ) have also been reported in leukopenic and thrombocytopenic dengue patients [173] (Table 1, Figure 2). These molecules are involved in leukocyte recruitment and inflammatory signaling, which can indirectly influence platelet activation and endothelial interactions [174]. Conversely, reduced levels of neutrophil‐derived proteins, including elastase 2, lactoferrin, and neutrophil gelatinase‐associated lipocalin (NGAL), as well as thrombospondin‐1, were observed in these patients. While the role of NGAL in dengue remains unclear, its association with leukocyte and platelet counts suggests a potential, albeit indirect, link to disease‐related hematological alterations [173].

The data presented here broadens our perspectives for their exploration in clinical practice as new biomarkers (Figure 1, Table 2) that can be investigated in the prognosis of dengue, thus contributing to the development of effective therapeutics to counteract disease severity. Furthermore, it is relevant to emphasize that only with effective combinations of classic biomarkers and potentially promising biomarkers (Figure 2), as herein described, can we foster the development of more effective therapeutic strategies, as dengue is a complex and multifactorial disease.

## 8 Final Considerations and Perspectives

This review highlighted classic and non‐classic biomarkers in dengue. In addition, accumulating evidence indicates that platelets are not only involved in hemostasis but also actively contribute to the modulation of immune responses and the maintenance of vascular integrity during dengue infection. We discussed the role of both classic and emerging platelet biomarkers in dengue, highlighting the available evidence regarding their association with disease severity, as well as their potential—albeit still limited in some cases—as therapeutic targets.

However, despite advances, important gaps remain to be further explored. Most biomarkers have been reported only in small‐scale observational studies, without standardization regarding collection timing, clinical criteria, or disease stage. Furthermore, most of the available research so far has evaluated biomarkers individually, whereas dengue pathophysiology indicates that only through multimarker panels integrating hemostatic, inflammatory, and endothelial axes will be possible to more accurately predict the disease clinical outcome. Additionally, there is a limitation due to the scarcity of functional and interventional studies in experimental models, which would provide evidence for the causal relevance of diagnostic targets that have, so far, been poorly explored.

Moreover, stratifying patients based on biomarker profiles can enable the development of individualized therapies, avoiding both unnecessary interventions in mild cases and critical delays in treating severe cases. Hence, integrating multimarker panels to the discovery of therapeutic targets has the potential to transform dengue care by enabling more specific prognostic approaches and patient‐tailored interventions, thereby reducing complications associated with thrombocytopenia, plasma leakage, shock syndrome, and even mortality. This review gathered the main markers studied for the early identification of disease severity in dengue infection and highlights them as possible therapeutic targets in disease development.

## Author Contributions

A.B.A.S.‐A.: participated in writing, review, editing, image creation (original draft, methodology, formal analysis, conceptualization); D.F.S.: writing (review and editing); J.C.D.: writing (review and editing); A.O.M.: imaging, writing, and editing; M.F.M.S.‐S. and E.D.H.: writing (review and editing); H.S.‐C.: writing (review and editing), formal analysis, and conceptualization. All authors read and approved the final manuscript.

## Funding

This work was supported by Coordenação de Aperfeiçoamento de Pessoal de Nível Superior (CAPES), CNPq | Instituto Nacional de Ciência e Tecnologia da Criosfera (INCT da Criosfera) (308732/2022‐7), and Conselho Nacional de Desenvolvimento Científico e Tecnológico (CNPq) (405394/2024‐1).

## Conflicts of Interest

The authors declare no conflicts of interest.

## Data Availability

This study is a narrative review based on previously published data. No new datasets were generated or analyzed; therefore, data sharing is not applicable.
